# No relevant modulation of TRPV1-mediated trigeminal pain by intranasal carbon dioxide in healthy humans

**DOI:** 10.1186/1129-2377-14-33

**Published:** 2013-04-10

**Authors:** Tim P Jürgens, Romy Reetz, Arne May

**Affiliations:** 1 Department of Systems Neuroscience, University Medical Centre Hamburg-Eppendorf, Martinistr. 52, Hamburg, D-20246, Germany

**Keywords:** Carbon dioxide, Trigeminal pain, TRPV1, Capsaicin, Headache

## Abstract

**Background:**

Nasal insufflation of CO_2_ has been shown to exert antinociceptive respectively antihyperalgesic effects in animal pain models using topical capsaicin with activation of TRPV1-receptor positive nociceptive neurons. Clinical benefit from CO_2_ inhalation in patients with craniofacial pain caused by a putative activation of TRPV1 receptor positive trigeminal neurons has also been reported. These effects are probably mediated via an activation of TRPV1 receptor - positive neurons in the nasal mucosa with subsequent central inhibitory effects (such as conditioned pain modulation). In this study, we aimed to examine the effects of intranasal CO_2_ on a human model of craniofacial pain elicited by nasal application of capsaicin.

**Methods:**

In a first experiment, 48 healthy volunteers without previous craniofacial pain received intranasal capsaicin to provoke trigeminal pain elicited by activation of TRVP1 positive nociceptive neurons. Then, CO_2_ or air was insufflated alternatingly into the nasal cavity at a flow rate of 1 l/min for 60 sec each. In the subsequent experiment, all participants were randomized into 2 groups of 24 each and received either continuous nasal insufflation of CO_2_ or placebo for 18:40 min after nociceptive stimulation with intranasal capsaicin. In both experiments, pain was rated on a numerical rating scale every 60 sec.

**Results:**

Contrary to previous animal studies, the effects of CO_2_ on experimental trigeminal pain were only marginal. In the first experiment, CO_2_ reduced pain ratings only minimally by 5.3% compared to air if given alternatingly with significant results for the main factor GROUP (F_1,47_ = 4.438; p = 0.041) and the interaction term TIME*GROUP (F_2.6,121.2_ = 3.3; p = 0.029) in the repeated-measures ANOVA. However, these effects were abrogated after continuous insufflation of CO_2_ or placebo with no significant changes for the main factors or the interaction term.

**Conclusions:**

Although mild modulatory effects of low-flow intranasal CO_2_ could be seen in this human model of TRPV-1 mediated activation of nociceptive trigeminal neurons, utility is limited as observed changes in pain ratings are clinically non-significant.

## Background

While efficacy of high-flow oxygen has been studied in several placebo-controlled studies on cluster headache [1,2] and recently in migraine as well [3,4], little is known on effects of carbon dioxide (CO_2_) on the craniofacial nociceptive system.

Phasic nasal insufflation of CO_2_ is used to elicit experimental trigeminal pain in humans [5] to record negative mucosal potentials [6] and chemosensory evoked potentials [7]. However, if given continuously, a significant habituation i.e. a decrease of CO_2_-induced pain can be observed after some minutes [8]. Likewise, repeated short stimuli of CO_2_ with high CO_2_ concentrations (more than 90% v/v) induce a rapid attenuation of negative mucosal potentials [6]. These results were translated into therapeutic utility by showing a reduction of post-dural puncture headache [9] and chronic cluster headache [10] upon prolonged inhalation of CO_2_. Preliminary data also show efficacy of nasal CO_2_ insufflation in migraine patients [11,12].

The putative mode of action is uncertain. As CO_2_ is a potent vasodilator, the first studies attributed possible therapeutic effects to the vasodilating properties on cerebral vessels. More recently, CO_2_ has been shown to be a powerful modulator of activated nociceptive trigeminal neurons [13]. Tzabazis and co-workers sensitized rat cheeks with capsaicin and insufflated CO_2_ or air nasally [14]. Nocifensive behaviour defined as facial and hind paw withdrawal to radiant noxious heat was significantly attenuated by higher CO_2_ flow rates of 0.8 l/min but not of 0.4 l/min CO_2_ or air. Based on additional pharmacological experiments the authors concluded that CO_2_ exerts its antinociceptive, respectively antihyperalgesic effects by activation of mucosal primary trigeminal afferents through a decreased mucosal pH within the nasal cavity.

These findings are well in line with the hypothesis that TRPV1 receptor positive trigeminal C and A delta fibres (which are activated by application of capsaicin) may play a relevant role in the pathogenesis of primary headaches such as migraine [15] although this concept has been challenged recently as TRPV1 receptor blockade was inefficient in *in vivo* models of migraine [16].

In summary, there are some clinical but also pre-clinical data that nasal instillation of CO_2_ could have some positive effect on acute headache. We therefore aimed to examine the modulatory efficacy of intranasal CO_2_ on experimental TRPV1-mediated trigeminal pain elicited by intranasal application of capsaicin in healthy volunteers to answer the following questions:

1. How painful is prolonged intranasal application of CO_2_ on a numerical rating scale at a flow rate of 1 l/min?

2. Does intranasally applied CO_2_ lead to relevant systemic changes of pH and pCO_2_ in capillary blood samples?

3. Does intranasal insufflation of CO_2_ modulate pain ratings after intranasal application of capsaicin?

## Methods

### Study design

We conducted a controlled randomized parallel-group study to investigate the effects of intranasal CO_2_ on TRPV1-mediated trigeminal pain in healthy volunteers. All participants provided written informed consent prior to inclusion into the study. Our study was approved by the local Ethics Committee (protocol number PV3814) and conformed to the Declaration of Helsinki.

### Subjects

Healthy volunteers were recruited among medical students at the Medical Faculty of Hamburg University (for epidemiological details see Table 1). Only subjects aged 18 years or above were considered. Exclusion criteria were: chronic pain in the medical history, acute craniofacial pain (such as tooth pain) within the last 4 weeks, intake of analgesics or triptans within the last 12 hours, respiratory tract infection within the last 2 weeks, bronchial asthma, chronic obstructive lung disease, respiratory insufficiency or other severe lung disease, allergy to capsaicin, pregnancy, lactation or participation in another clinical trial within the last 3 months.

**Table 1:** Reporting of epidemiological details for the entire cohort and the corresponding subgroups

	**All**	**CO**_**2**_	**Air**	**Statistics**
Mean age (SEM)	24.8 years (±0.5)			
		24.5 (±0.6)	25.1 years (±0.7)	t(46) = -0.636; p = 0.528
Gender ratio (male:female)	24:24			
		12:12	12:12	χ2(1, n = 48) = 0.000; p = 1.000

Results of unpaired t-tests are considered significant with p < 0.05.

### Experimental design

#### Pilot study on the effects of nasal CO_2_ insufflation

**Figure 1:**
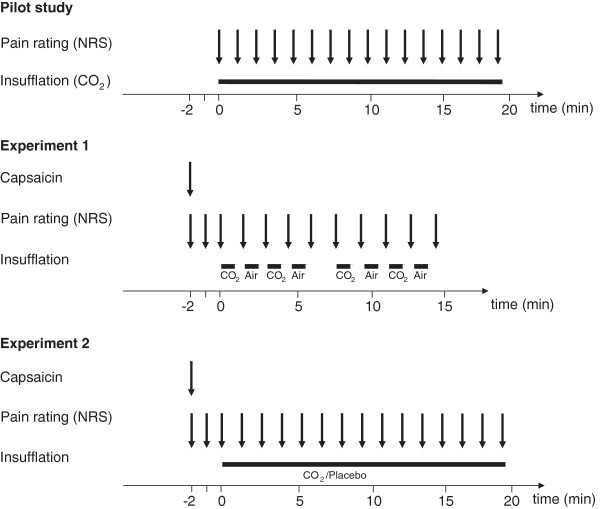
**Experimental setup.** NRS: numerical rating scale.

In a pilot study (Figure 1) designed to quantify possible side effects and pain evoked by CO_2_ insufflation in particular, 20 subjects received intranasal CO_2_ (1 l/min) for 18:40 minutes and rated their pain on a numeric rating scale (NRS) from 0 (no pain) to 10 (worst imaginable pain) every 80 seconds. To assess possible systemic changes in pH and CO_2_ levels, pH and pCO_2_ were additionally determined in 10 patients by a capillary blood gas analyses. This was taken from the earlobe before nasal installation of CO_2_ started and immediately after the last pain rating while the participants were still exposed to CO_2_.

#### Experiment 1: alternating nasal application of CO_2_ and air after nociceptive stimulation with intranasal capsaicin

In the main study (Figure 1), 48 healthy volunteers received intranasal CO_2_ (1 l/min) and air (1 l/min) alternatingly. If volunteers had already participated in the pilot study, both experimental sessions were separated by at least 6 weeks. Again, subjects rated the magnitude of pain verbally on a numerical rating scale (NRS) ranging from 0 (non-painful) to 10 (worst imaginable pain).

Initially, participants received one puff of capsaicin spray (200 μg per puff, custom formulation supplied by the local pharmacy, for details see below) into the left nostril to trigger TRPV1-mediated trigeminal nociception. After three initial pain ratings on a NRS every 60 seconds (Figure 1), CO_2_ and air were insufflated for 60 sec each in an alternating fashion via a nasal cannula. During an interval of 20 sec between exposure to either CO_2_ or air, no gas was applied and the participant was asked to rate the resulting pain on a NRS. After 4 cycles, insufflation was interrupted for 1 min to allow participants to remove excessive nasal discharge. However, subjects were asked not to blow their noses to avoid early removal of the intranasal capsaicin. Thereafter, 4 identical cycles of CO_2_, respectively air were given and subjects were asked to rate the pain on the NRS.

#### Experiment 2: nasal application of CO_2_ or placebo after stimulation with intranasal capsaicin

In the subsequent second experiment separated from the first experiment by at least 4 weeks all participants from the first experiment were randomly allocated to receive either CO_2_ (1 l/min) or placebo in 2 subgroups of 24 subjects each.

Again, participants received 1 puff of capsaicin spray into the left nostril. Pain ratings on a NRS were noted every 60 sec over the entire duration of this experiment (Figure 1). After 120 sec, participants received either continuous nasal insufflation of CO_2_ for a total of 18:40 min via a nasal cannula or placebo (nasal cannula alone without any gas insufflation) and rated the pain on the NRS as in the pilot study.

### Insufflation of CO_2_ and air

Medical grade CO_2_ (TMG, Krefeld, Germany) was administered from compressed gas cylinders with a volume of 10 l. By using a combined pressure reducing valve (200 mbar/4.5 mbar) and flowmeter (Gloor, Burgdorf, Switzerland) a constant flow of 1 l/min was maintained. Medical grade compressed air was obtained from the hospital gas reticulation system and delivered at a flow of 1 l/min by a flow meter (Dräger, Lübeck, Germany). Nasal cannulae (Dahlhausen, Köln, Germany) were used to apply both gases locally into both nasal cavities.

### Breathing technique

As CO_2_ should only be insufflated nasally and not inhaled due to safety concerns, all subjects were trained before the first experiment. CO_2_ was applied bilaterally into the anterior nasal cavity, where highest mucosal responsiveness of evoked potentials to short pulses of CO_2_ could be found [17], namely at the anterior septum as compared to more posterior parts. They were instructed to breathe-in through the mouth und exhale through the nose. Before each experiment they were reminded to adhere to these instructions.

### TRPV1 activation with capsaicin

One puff of a nasal spray containing 200 μg of capsaicin (prepared from 1.42 ml capsaicin liquid extract in 10 ml of refined sesame oil) was applied to the left nostril with the head in a stooped position to avoid contamination of the pharynx. Participants were allowed to remove excessive nasal discharge by soaking cotton swaps, but were asked not to blow their noses to avoid early removal of intranasal capsaicin.

### Capillary blood gas analysis

Blood gas analyses were collected in from the earlobe in capillary tubes after pretreatment with an ointment containing 5% benzyl nicotinate (supplied by the hospital pharmacy) for 10 mins. Immediately afterwards samples were tested with a blood gas analyzer (ABL5, Radiometer Medical, Brønshøj, Denmark). The manufacturer’s reference values for capillary blood gas analysis were as follows: pH 7.35- 7.45; pCO_2_ males: 35-48, females: 32-45.

### Data evaluation and statistics

Descriptive statistics are given as mean values and standard errors of the mean. Differences on mean values were either examined by paired t-tests or by means of a repeated measures analysis of variance (ANOVA). For analysis of pain ratings over time in the pilot study, the factor TIME (pain ratings 1-15) was used, for other comparisons of pain ratings a two-way repeated measures ANOVA with the factors TIME (corresponding pain ratings) and GROUP (CO_2_ or air). Comparison of categorical data (gender) was done by means of the chi square test. In all tests p values <0.05 were considered significant.

Bivariate correlations were calculated by Pearson’s correlation analysis with p < 0.05 regarded as significant. All analyses were done with SPSS 20 (IBM, Amonk, NY, USA).

## Results

### Nociceptive properties of intranasal CO_2_

**Figure 2:**
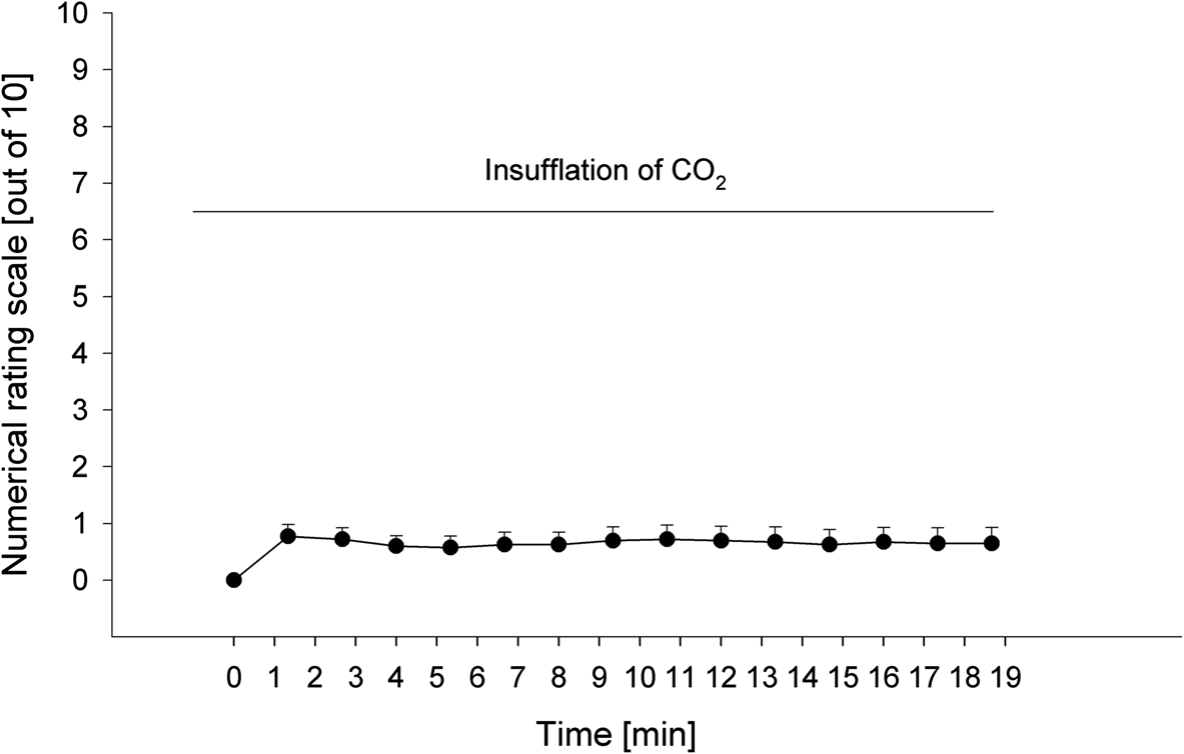
**Nociceptive properties of nasally insufflated CO**_**2**_**.** Pain ratings on a numerical rating scale from 0 to 10 during nasal insufflation of CO_2_ with a flow of 1 l/min for 20 min. Error bars are given as standard error of the mean.

When CO_2_ was given exclusively to 20 healthy subjects (10 male, 10 female, mean age 24.4 ± 0.7 years, range 21-35 years), mean pain ratings over time were 0.6 (± 0.06) out of 10 on the NRS. Pain ratings reached their maximum after 1 min of inhalation with 0.8 (±0.21) out of 10 on the NRS (see Figure 2). A repeated-measures ANOVA for the factor TIME (pain ratings 1 to 15) yielded a significant effect (F_14,266_ = 2.039; p = 0.001). Seven (35%) of the participants indicated that they had not perceived any pain at all.

**Figure 3:**
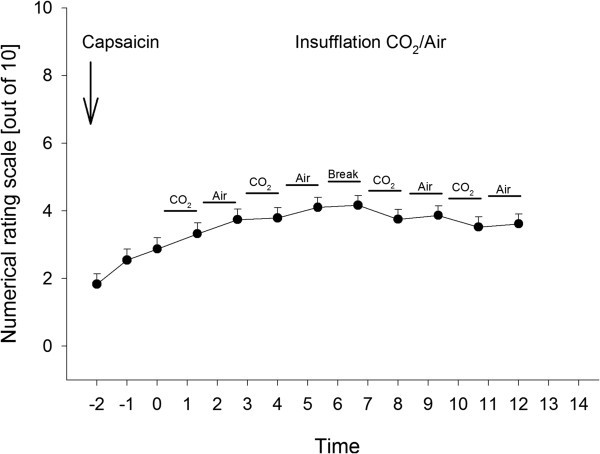
**Alternating insufflation of CO**_**2 **_**and air after administration of capsaicin.** Pain ratings on a numerical rating scale from 0 to 10 during nasal insufflation of CO_2_ with a flow of 1 l/min or compressed air for 60 sec each followed by an interval of 20 sec to change gas supply and pain rating after intranasal application of capsaicin. After 2 cycles of CO_2_ and air each a break of 60 sec was made to allow subject to remove nasal discharge due to intranasal capsaicin application. Time is given in minutes. Error bars are given as standard error of the mean.

**Figure 4:**
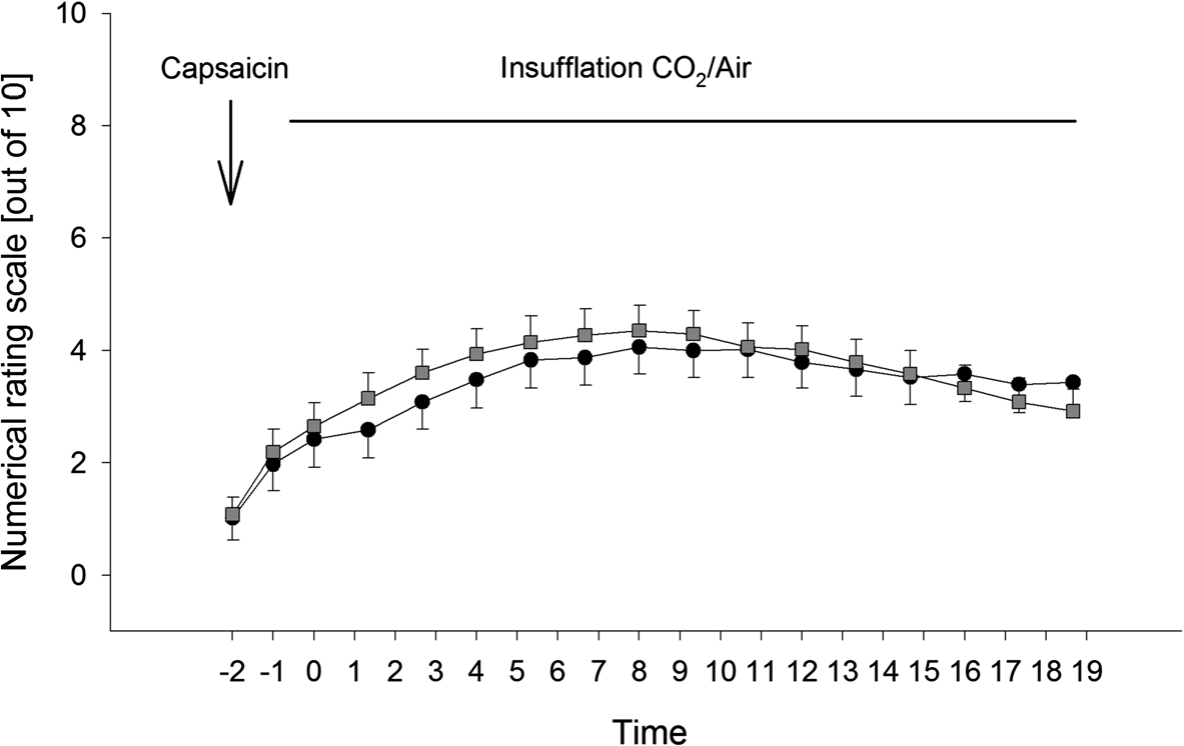
**Continuous insufflation of either CO**_**2 **_**or air after administration of capsaicin.** Pain ratings on a numerical rating scale from 0 to 10 before and during nasal insufflation of CO_2_ (black dots) with a flow of 1 l/min or compressed air (grey boxes) for 19 minutes after intranasal application of capsaicin. The time is given in minutes. Error bars are given as standard error of the mean.

If subjects accidentally inhaled CO_2_ nasally or started talking during the training phase, they mostly complained of a highly unpleasant irritation spreading from the nasal cavity into the nasal sinus and the pharynx which led to interruption of the training.

Net changes of pCO_2_ levels (difference between pCO_2_ after – pCO_2_ before insufflation) did not correlate with corresponding pain ratings in the pilot study (Pearson’s bivariate correlation: p > 0.05).

### Modulatory effects of *alternating* insufflation of CO_2_ and air on TRPV1-mediated nociceptive trigeminal activation

For epidemiological details see Table 1. Mean values for all pain ratings after insufflation of CO_2_ (3.6 ± 0.3) and air (3.8 ± 0.3) respectively differed significantly (t(47) = -2.107; p = 0.041). Likewise, a repeated-measures ANOVA for the factors TIME (pain ratings for insufflation 1 to 4) and GROUP (CO_2_ or air) yielded no significant main effect for TIME (F_1.3,63.3_ = 2.039; p = 0.153), but significant results for both the main factor GROUP (F_1,47_ = 4.438; p = 0.041) and the interaction term TIME*GROUP (F_2.6,121.2_ = 3.3; p = 0.029). Mean values are given in Figure 3.

### Modulatory effects of *continuous* insufflation of CO_2_ or air on TRPV1-mediated nociceptive trigeminal activation

Age and gender distribution did not differ significantly between the randomly assigned subgroups receiving either CO_2_ or air in experiment 2 (for further details see Table 1). Mean values for each pain rating are given in Figure 4.

A repeated-measures ANOVA with the factors TIME (pain ratings 1 to 17) and GROUP (CO_2_ or air) yielded a significant main effect for TIME (F_2.1, 98.4_ = 20.119; p < 0.001), but not for the factor GROUP (F_1,46_) = 0.089; p = 0.767) or the interaction term TIME*GROUP (F_2.1, 98.4_ = 0.624; p = 0.548).

There was no baseline difference defined as comparison of the first pain rating between the CO_2_ and the air group after administration of capsaicin and before the first insufflation (CO_2_: 1.0 ± 0.4_;_ air: 1.1 ± 0.3; t(46) = 0.15; p = 0.902).

### Side effects of treatment

No subject terminated the study early or complained about side-effects apart from unpleasantness or mild pain upon nasal insufflation of CO_2_. Most notably, no patient complained about dyspnea or other clinical side effects. After 20 min of CO_2_ insufflation, a significant shift of capillary pH and capillary pCO_2_ readings could be found. pH decreased and pCO_2_ levels increased significantly (see Table 2 for further information). However, these changes were well within the normative range. Apart from a parasympathetic activation involving lacrimation and rhinorrhea, intranasal capsaicin or insufflation of CO_2_ induced no relevant side-effects.

**Table 2:** Reporting of safety data for 10 patients taking part in to pilot study and the corresponding statistical tests (paired t-tests, considered significant with p < 0.05)

	**Before CO**_**2**_**insufflation**	**After CO**_**2**_	**Statistics**
pH	7.43 (±0.01)	7.41 (±0.01)	t(9) = 3.21; p = 0.011
pCO_2_	35.0 (±1.4)	37.3 (±1.0)	t(9) = -3.15; p = 0.012

## Discussion

Contrary to previous animal studies, the effects of intranasally insufflated CO_2_ on experimental trigeminal pain were marginal. Application of CO_2_ alone resulted in mild pain and a significant but clinically irrelevant change in capillary pCO_2_ and pH levels. After nociceptive stimulation of the first trigeminal branch with intranasal capsaicin (as a model of TRPV1-mediated craniofacial pain with central sensitization), alternating application of CO_2_ reduced pain ratings only minimally compared to a sham paradigm with air, but this effect was abrogated during continuous insufflation of CO_2_.

### Intranasal application of capsaicin as a human model of trigeminal nociceptive activation

Capsaicin (8-Methyl-N-vanillyl-trans-6-nonenamide) increases release of substance P and simultaneously blocks its reuptake exerting its effects by activation of the TRPV1 receptor [18]. This leads to an activation of the trigemino-vascular system with enlargement of the internal carotid artery [19] if applied intracutaneously into the skin innervated by the ophthalmic division (V1) of the trigeminal nerve but not when applied to the skin innervated by the mandibular (V3) branch or the forearm. The nasal mucosa is innervated by branches of the ethmoidal (V1) and the maxillary (V2) nerve [20] with neurons containing predominantly substance P and - to a much lesser extent - CGRP [21]. Noxious stimulation with capsaicin applied to the nostril causes long-lasting discharges of afferent nerve fibres of the ethmoidal nerve in guinea pigs [22]. In another study also in guinea pigs, expression of the immediate-early gene c-fos after intranasal capsaicin was highest in the trigeminal complex with the subnuclei caudalis and interpolaris [23]. Interestingly, the majority of cells which innervate the dura show a co-localisation of TRPV1 receptors and CGRP [24,25].

It has been shown in *in vitro* experiments with slices of rat trigeminal nucleus caudalis [26] that activation of TRPV1 receptors on nociceptive trigeminal afferents causes release of CGRP. CGRP itself does not activate meningeal afferents but is thought to facilitate nociceptive transmission in the spinal trigeminal nucleus via presynaptic modulation of other primary afferents rather than direct effects on second-order neurons in the spinal trigeminal nucleus [27,28]. A relevant role in migraine pathophysiology is supported by the observation that migraine attacks can be aborted by administration of CGRP receptor antagonists [29,30]. According to Lambert and colleagues the TRPV1 receptor antagonist SB-705498 does not suppress activation in the trigeminal nucleus caudalis in cats following electrical and mechanical stimulation of nociceptive meningeal and facial afferents. However, sensitisation induced by inflammatory soup was significantly attenuated by SB-705498 [31]. In contrast, Summ and co-workers challenged a relevant role of TRPV1 receptors in migraine pathophysiology as blockade of TRPV1 receptors did neither modulate experimentally induced cortical spreading depression nor neurogenic dural vasodilation or nociceptive transmission in the trigemino-cervical complex in rats [16].

Regardless of whether TRPV1 receptor positive V1 neurons are indeed involved in migraine pathophysiology or not, they have been associated with other craniofacial pain syndromes such as dental pain where an upregulation of TRPV1 expression in rat trigeminal ganglia was observed in a model of lipopolysaccharide-induced pulpitis [32]. We note that we did not strive for a specific model such as migraine headache but a robust model of craniofacial pain including TRPV1 activation affecting the first trigeminal branch. In our sample, participants showed a transient and significant increase in pain ratings after nasal application of capsaicin which is in line with other studies showing a subsequent and temporary regional sensitization [14,33]. Pain ratings peaked after 8-9 min which is congruent with an animal study on neuronal activity in the rat nucleus caudalis [34]. After application of capsaicin to the eye and the tongue a delayed activity peaking also after 4-6 min was found. Thus, intranasal application of 200 μg capsaicin is a potent model for stimulation of TRPV1 positive nociceptive neurons of the first trigeminal branch.

### Characteristics, tolerability and safety of intranasal insufflation of CO_2_

CO_2_ insufflation was rated as mildly painful by 65% of patients. We found systemic changes in capillary pH and pCO_2_ levels but no relevant alterations beyond the normative range and conclude that nasal application at 1 l/min is safe. Insufficient delivery of CO_2_ into the nasal cavity seems unlikely as changes of pCO_2_ levels after nasal insufflation did not correlate with pain ratings in our pilot study.

Our attempts to mimic CO_2_ induced unpleasantness respectively pain with vaporized ammonia to establish a robust sham condition were in vain (data not shown). Ammonia induced a more stabbing and - at higher concentrations - unbearable pain in the nasal cavity with variable pain thresholds. The unpleasantness of *intranasal* CO_2_ in our sample implies that true blinding is not feasible at flow rates of 1 l/min and above as most patients will notice an unpleasant or painful perception. As clinical effects were negligible in our sample, potential sources of bias such as a relevant placebo effect are unlikely.

At lower *intranasal* flow rates of 0.6 l/min, blinding seemed to be less problematic in prior studies. Casale et al. [35] reported that 80% of patients with seasonal allergic rhinitis did not notice nasal stinging or burning during nasal *insufflation* of CO_2_ at 0.6 l/min for only 1 minute – similar to the setup used in the therapeutic studies in migraine patients by Spierings [11,12]. *Inhalation* of CO_2_ was less well tolerated raising doubts about effective blinding in prior studies reporting efficacy of inhaled CO_2_.

### Modulatory effects of CO_2_ insufflation on nociceptive trigeminal activation

The antihyperalgesic efficacy of CO_2_ in our study was small and reached statistical significance only when CO_2_ and placebo were given in an alternating fashion within the same subject (experiment 1) – as opposed to application of either CO_2_ or air only in experiment 2. It may be easier for the participants to sense a subtle analgesic or anti-hyperalgesic efficacy of CO_2_ if it is given in a contrasting fashion with air as placebo.

As shown by Tzabazis and colleagues in a rat model [14], nasal insufflation of CO_2_ with flow rates of 0.8 l/min attenuated nocifensive behaviour after sensitization with capsaicin unlike lower flow rates of CO_2_ (0.4 l/min) or air. In the nasal mucosa CO_2_ decomposes into protons and carbonate catalyzed by mucosal carbonic anhydrase and activates TRPV1- and ASIC- positive neurons by proton accumulation. Subsequent, central inhibitory effects are proposed such as a widespread inhibition of afferent trigeminal input though inhibitory interneurons, trans-segmental inhibitory control circuits or conditioned pain modulation.

Vause and colleagues showed that incubation of cultured trigeminal ganglions with CO_2_ or capsaicin resulted in an acidification of culture medium and a consecutive nociceptive activation with CGRP release [13]. Similarly, CO_2_ attenuated CGRP release by pretreatment with capsaicin if cultured under isohydric conditions which prevents extracellular but allows intracellular acidification.

Tzabazis and co-workers observed less intense and only short-lived antinociceptive or antihyperalgesic effects of CO_2_ insufflation on non-sensitized skin as compared to air insufflation [14]. These findings strongly argue in favour of activity-dependent effects of CO_2_, so that efficacy could have been better in patients with chronic craniofacial pain. Alternatively, CO_2_ inhalation with a potentially different locus of action could represent a more powerful alternative although tolerability seems to limit feasibility [9,10].

In summary, intranasal insufflation of CO_2_ exerts antihyperalgesic effects in animal models but resulted in only minor clinical effects in our human model of trigeminal pain elicited by activation of nociceptive TRPV1 receptors in healthy volunteers. These moderate effects question the clinical utility of intranasal CO_2_ in TRPV1-mediated pain at flow rates of 1 l/min.

### Clinical efficacy of CO_2_

Marcussen and Wolff successfully treated aura symptoms in migraine patients termed as “vasoconstrictor symptoms” by inhalation of 10% CO_2_ in either air or oxygen for 5 min [36]. Likewise, Sikh and Agarwal exposed 40 patients with post-dural puncture headache to 5.6% CO_2_ mixed with oxygen for 10 min daily which was repeated up to 2 times if the headache was not relieved [9]. After 3 days, 98% of the patients reported relief compared to 58% of the oxygen only control group. Despite these impressive clinical results on primary and secondary headaches, patients inhaled much higher concentrations of CO_2_ suggesting a potentially different mode of action. In addition, translating these clinical results to our human model is difficult as the role of the TRPV1 receptor in migraine pathophysiology has been challenged [16]. Furthermore, some studies yielded contrasting results. Engel reported that inhalation of 10% CO_2_ provoked a headache attack or increased headache intensity in 18 of 40 subjects with mainly posttraumatic headache and migraine. CO_2_-induced headache attacks or aggravation were less intense than headaches triggered by histamine and adrenaline [37]. Likewise, Hannerz and Jogestrand reported that ipsilateral pain could be elicited in patients with active episodic cluster headache during inhalation of 6% CO_2_ for 6 min [38].

At present, a firm conclusion on the clinical efficacy of CO_2_ in primary and secondary headaches is difficult despite promising data from animal experiments. Trials on the efficacy of inhaled CO_2_ in various headache syndromes are relatively old and yielded ambiguous results. Evidence for the efficacy of intranasal CO_2_ in migraine has been published in preliminary form.

## Conclusions

Only mild modulatory effects of intranasal insufflation of CO_2_ at flow rates of 1 l/min could be seen in a human model of TRPV1 mediated activation of nociceptive trigeminal neurons which is in line with previous studies. While application was safe, clinical utility at low flow rates was limited in our model as the therapy is uncomfortable and changes in pain ratings are therapeutically irrelevant.

## Competing interests

TPJ and RR: no relevant conflicts of interest, AM: has received unrestricted scientific grant support from Linde Gas (RealFund; http://www.linde-healthcare-realfund.com).

## Authors’ contributions

TPJ conceived of the study, participated in the conception and design, performed the statistical analysis, interpreted and discussed the data and drafted the manuscript. RR participated in the design of the study and data acquisition and helped in the interpretation of it. AM participated in the conception and design of the study, interpreted and discussed the data and drafted the manuscript. All authors read and approved the final manuscript.
